# The Liberation Procedure Decision-Making Experience for People With Multiple Sclerosis

**DOI:** 10.1177/2333393614551413

**Published:** 2014-10-08

**Authors:** Cynthia L. Murray, Michelle Ploughman, Chelsea Harris, Stephen Hogan, Michelle Murdoch, Mark Stefanelli

**Affiliations:** 1 Memorial University, St. John’s, Newfoundland and Labrador, Canada; 2 Coalition of Persons With Disabilities, St. John’s, Newfoundland and Labrador, Canada

**Keywords:** decision making, health care, users’ experiences, hermeneutics, lay concepts and practices, lived experience, multiple sclerosis (MS), phenomenology, research, qualitative

## Abstract

Despite the absence of scientific evidence demonstrating the efficacy of the “liberation procedure” in treating multiple sclerosis (MS), thousands of MS patients worldwide have undergone the procedure. The study objective was to explore the experience of liberation procedure decision making for individuals with MS. Fifteen adults in Newfoundland and Labrador, Canada, each participated in an in-depth interview. The data analysis revealed three groups of people: “waiters,” “early embracers,” and “late embracers.” Using van Manen’s hermeneutic phenomenological approach, we identified three themes each in the stories of the early and late embracers and four themes in the waiters’ stories. A characteristic of the late embracers and waiters was skepticism, whereas desperation set the embracers apart from the waiters. With a deeper understanding of the experience, nurses can be more attuned to the perspectives of MS patients while helping them make informed decisions about undergoing the liberation procedure.

Multiple sclerosis (MS) is an inflammatory, demyelinating disease of the central nervous system with an unknown etiology that affects more than two million individuals worldwide (Multiple Sclerosis International Federation, 2013). Research on the natural history of MS paints a picture of a disease with different trajectories (Debouverie, Pittion-Vouyovitch, Louis, & Guillemin, 2008). However, 80% of MS patients have a walking impairment within 10 to 15 years of disease onset (Souza et al., 2010), and 50% of these patients require ambulatory assistance in another 5 years (i.e., within 15–20 years of disease onset), even though disease progression might be slowing down (Tremlett, Zhao, Rieckmann, & Hutchinson, 2010). The risk of all-cause mortality in MS patients is two to three times that in the general population (Kingwell et al., 2012). The global economic and human costs of MS are high. In the United States alone, the total annual expenditures attributable to MS are estimated at US$billion (National Multiple Sclerosis Society [NMSS], 2013a).

Disease-modifying therapies diminish the frequency and severity of relapses, decelerate the progression of disability, decrease the number of brain lesions, and enhance the quality of life of MS patients (Goodin et al., 2002). However, there is no cure for MS (NMSS, 2013b). Consequently, MS patients often resort to complementary and alternative medicine (CAM) in the hope of obtaining an effective treatment (Olsen, 2009). In fact, despite the lack of conclusive empirical evidence supporting the usefulness of several CAM modalities for the treatment of MS (Bowling, 2011; Namaka et al., 2008), this patient population has a widespread pattern of CAM use (Stoll, Nieves, Tabby, & Schwartzman, 2012). In the past, the MS community at large has eagerly tried controversial and unsubstantiated remedies, such as bee-venom therapy (Wesselius et al., 2005).

In 2009, the news of a novel theory on the nature of MS, which was proposed by the Italian physician and researcher Paolo Zamboni, captured the attention of international media outlets. Zamboni reported that MS might be the result of chronic inadequate venous flow in the central nervous system and can be alleviated by a procedure that was coined by him as the “liberation procedure” (Zamboni, Galeotti, Menegatti, Malagoni, Gianesini, et al., 2009; Zamboni, Galeotti, Menegatti, Malagoni, Mascoli, et al., 2009; Zamboni, Galeotti, Menegatti, Malagoni, Tacconi, et al., 2009). On November 21, 2009, the Canadian Television Network (CTV) aired a special program about the liberation procedure in its award-winning documentary show called W5 (Favaro & St. Philip, 2009; “Kevin Newman,” 2014).

Since 2009, in the absence of scientific evidence demonstrating the efficacy of the liberation procedure for the treatment of MS, thousands of MS patients worldwide have undergone the new experimental procedure (O’Neill, Mazanderani, & Powell, 2012; Pryse-Phillips, Stefanelli, Murphy-Peddle, & Barrett, 2013). During the exodus of MS patients to the small number of countries that offer the unproven and radical liberation procedure, such as Costa Rica and Poland, authors of professional publications and the mass media reported on the fatal complications of the procedure and issued medical warnings (Alphonso, 2010; American Neurological Association, 2010; Mansour et al., 2010). Nevertheless, many individuals who could either afford or raise funds for the expensive procedure (i.e., up to tens of thousands of dollars for the procedure alone) traveled abroad to undergo the procedure based on Zamboni’s highly speculative theory, which is called chronic cerebrospinal venous insufficiency (CCSVI; Mansour et al., 2010).

MS patients give several reasons for seeking unconventional treatments. Besides disillusionment with mainstream medication and medical care (Hussain-Gambles & Tovey, 2004), we noted that negative characteristics of health care provider–patient relationships and communications were nestled among the most common reasons mentioned in the literature. For example, in explaining the rationale behind their decision to undergo CAM, MS patients cited poor patient–physician relationships (Mews & Zettl, 2012), brief medical consultations (Schwarz, Knorr, Geiger, & Flachenecker, 2008), and the lack of understanding and support from health care providers (Hussain-Gambles & Tovey, 2004). Moreover, we identified calls in the literature for health care practitioners and researchers to communicate better with patients and the public about CAM in general (Gaylord & Mann, 2007) and the liberation procedure in particular (Pullman, Zarzeczny, & Picard, 2013); this would help the former in gaining insights on the perspectives of MS patients and facilitating informed patient decision making.

Given the high regard for nurses among the general population (Swift, 2013), they play an important role in patient decision making in general (Say, Murtagh, & Thomson, 2006). With a deeper understanding of the experience of decision making regarding the liberation procedure for MS patients, nurses and other health care practitioners can be more attuned to the perspectives of MS patients while helping them in making informed decisions about the procedure. To our knowledge, however, there are no published qualitative studies in this area till date; therefore, we set out in this study to answer the following research question:

**Research Question 1:** What is the experience of liberation procedure decision making for persons living with MS?

## Method

Given the abovementioned aim of our study, hermeneutic phenomenology was considered an appropriate methodology for this research. The aim of using hermeneutic phenomenology is to gain a better understanding of the meaning or significance of human lived experiences (van Manen, 1990), which generates practical knowledge and fosters reflective clinical practice (Bergum, 1991).

### Setting and Participants

We conducted this study on the east coast of Canada, in the province of Newfoundland and Labrador (NL). The prevalence of MS in Canada is among the highest in the world (Beck, Metz, Svenson, & Patten, 2005; Marrie, Yu, Blanchard, Leung, & Elliott, 2010). In 2005, researchers calculated the prevalence of MS in the Atlantic region of Canada, which includes NL, at 350 per 100,000 individuals (Beck et al., 2005).

The inclusion criteria for this study were English-speaking men and women aged 19 years or older, with a diagnosis of MS, and who considered whether or not to undergo the liberation procedure. After ethical approval from the Health Research Ethics Board in St. John’s, NL, we recruited participants through MS outpatient services. Health care providers or group facilitators in these clinical and support group settings informed potential participants about the study. The second author contacted individuals who were interested in participation, explained the study again to these potential participants, and arranged interviews with those who agreed to take part. A total of 15 adults (10 women and 5 men) were eventually included. We expected more women than men among the participants because the women-to-men MS ratio is approximately 3:1 (Marrie et al., 2010; Sloka, Pryse-Phillips, & Stefanelli, 2005).

The participants were divided into three groups on the basis of their decisions regarding the liberation procedure. The first group included seven participants (five women, two men; mean age, 46 years; *SD*, 11 years) who immediately decided to undergo the liberation procedure. Three of these participants had a high school diploma, whereas four had received some postsecondary education. The second group included two participants (one woman, one man; mean age, 39 years; *SD*, 3 years) who decided to undergo the liberation procedure later; one had a high school diploma, and the other held a university degree. The third group included six participants (four women, two men; mean age, 52 years; *SD*, 9 years) who decided against undergoing the liberation procedure; the two men had received some postsecondary education, and the four women held a university degree.

### Data Collection

We collected data by conducting one face-to-face interview with all participants except one; we accommodated the wishes of this participant by conducting the interview over the telephone. The second author digitally recorded and conducted all interviews at a time and place chosen by each participant. As it turned out, all of the face-to-face interviews took place in the participants’ homes. The researcher obtained written informed consent before beginning each interview, including the telephone interview. Specifically, the researcher reviewed all sections of the consent form (e.g., purpose of the research, description of study procedures, benefits and risks, privacy and confidentiality, the right to withdraw, permission to audio record the interviews) with each participant and answered their questions. Then, the researcher collected sociodemographic and MS-related medical information, including age, level of education, type of MS, length of time since diagnosis, and use of disease-modifying therapies, to describe the sample and help in interpreting the findings.

The second author initiated the interview with a broad, open-ended question that allowed the participant to talk freely about her or his experience with MS. Subsequently, the researcher posed follow-up, open-ended questions to explore the participant’s experience with the liberation procedure. The follow-up questions included the following: How did you find out about the liberation procedure? What do you think about the liberation procedure? How did you decide to have the liberation procedure? How did you decide to not have the liberation procedure? Who have you told about your decision to have/not have the procedure? How did they react?

Although an interview guide that included possible questions for the participants was developed, the interviews were flexible in that the researcher asked spontaneous, open-ended questions to engage in a dialogue with the participant and elicit her or his unfettered story about the liberation procedure decision-making experience. The researcher used prompts such as “Could you please tell me more about that?” and “How did that make you feel?” to get the participant to elaborate on certain aspects of her or his story and obtain deeper and richer descriptions. The interviews lasted between 60 and 90 min. We stopped recruiting participants and collecting data once a good phenomenological gestalt was reached, as indicated by an “inner unity in the text” (Kvale, 1983, p. 186), and once additional interviews only provided redundant information. Data were collected between May 2011 and April 2012.

### Data Analysis and Rigor

We used the hermeneutic phenomenological approach of van Manen (1990) in this study. This approach involved a nonlinear interplay between six research activities: (a) turning to the nature of lived experience, (b) investigating experience as we live it, (c) reflecting on essential themes, (d) writing and rewriting, (e) maintaining a strong and oriented relation, and (f) balancing the research context by considering parts and the whole (van Manen, 1990).

Specifically, the data analysis unfolded in the following manner. Initially, a transcriptionist transcribed the recorded interviews verbatim and the first author checked the transcripts for accuracy. We used the computer software program NVIVO 10 (Qualitative Solutions Research International, 2012) to organize the data. All members of the research team independently read, reread, and reflected on all of the transcripts. The research team included one researcher who was proficient in hermeneutic phenomenological methodology and other researchers who were experts in MS or disabilities. Two other members of the research team were research assistants. Drs. Murray and Ploughman independently identified preliminary themes in the transcripts by using a selective approach and moving back and forth between elements of the text and the whole text.

After the initial analysis, we held research team meetings to discuss the preliminary themes. On the basis of phenomenological reflection using the four existentials (i.e., lived body, lived time, lived space, and lived human relation), the first author, Cynthia Murray, took the lead in writing, rewriting, and refining the themes identified. For instance, Murray further elucidated, via phenomenological refection, that some of the participants perceived their bodies as unstable (lived body) and time as being of the essence (lived time), whereas others perceived the opposite. We reached a unanimous agreement on the themes through discussion.

To attain rigor, we used verification strategies (Meadows & Morse, 2001; Morse, Barrett, Mayan, Olson, & Spiers, 2002). The specific verification strategies used included investigator responsiveness, methodological coherence, concurrent data collection and analysis, and sampling sufficiency. We remained open and listened to the data. We put on hold our prior knowledge about the phenomenon of interest (Meadows & Morse, 2001) and abandoned ideas that were not supported by the data. Our research question, methods, and analytical procedures were congruent with each other, and we maintained an audit trail of the research process. We included researchers and clinicians with expertise in MS, disabilities, or qualitative methodology on the research team. Data collection and analysis were concurrent. We continued to collect data and recruit participants, with first-hand knowledge and lived experiences of the phenomenon under investigation, until redundancy occurred and there was a gestalt in the text. All of the team members ensured that the findings fit the data.

## Findings

The data analysis revealed two distinct main groups of people: “embracers of the liberation procedure” (*n* = 9) and “waiters” (*n* = 6). Depending on the timing of their decision to undergo the procedure, we subdivided the embracers of the liberation procedure into “early embracers” and “late embracers.” Next, we discuss each main group and subgroup in succession.

### Embracers of the Liberation Procedure

All embracers of the liberation procedure endorsed Zamboni’s treatment. However, the timing of their endorsement varied: Seven individuals decided to undergo the procedure immediately, if possible, while two followed a longer and more complex decision-making path before reaching their ultimate decision. We describe these early and late embracers in the following sections.

#### Early embracers

We discovered three themes in the stories of the early embracers. Specifically, the themes were as follows: jumping at a promising opportunity to get better, desperately trying to ascend to a better quality of life, and having no regrets (see Table 1).

**Table 1.** Themes Identified in the Participants’ Stories of Decision Making Regarding the Liberation Procedure.

Participant group	Theme
Early embracers	Jumping at a promising opportunity to get better
	Desperately trying to ascend to a better quality of life
	Having no regrets
Late embracers	Greeting the liberation procedure with skepticism
	A last-ditch effort
	Risking everything
Waiters	Putting the liberation procedure under the microscope
	Skeptically refusing to jump or stay on the bandwagon
	Not wanting to rock the boat
	Waiting for credible research results

#### Jumping at a promising opportunity to get better

While watching the W5 program titled “The Liberation Treatment: A Whole New Approach to MS” (Favaro & St. Philip, 2009), the early embracers felt a surge of hope and excitement. The program (Favaro & St. Philip, 2009) roused them and led them to believe that a new treatment or cure for MS had been found. For the early embracers, the new approach to MS was so convincing that they jumped on it, as depicted in the following excerpt:

I saw it on TV actually. It seemed like promising news to everybody . . . They were telling a big breakthrough for MS . . . I said, “Finally there’s a cure for MS.” . . . And all of a sudden, boom, I said, “I’m going to jump on it then ‘cause everyone . . . got feelings and that back in their hands, their legs, their feet, and . . . some people are actually getting out of wheelchairs.” . . . It’s bringing a lot of hope to people.

Another participant stated the following:

Dr. Zamboni said that his wife was just starting to show the signs of MS and . . . [that] the procedure . . . stopped [it] . . . right in its tracks. [I said,] “I’m going to walk!” . . . The more I heard [the W5 show], the more excited I got . . . I was saying [I want] ten pairs of high-heeled shoes . . . [and] I want to dance until flames . . . [come] out of them. I was right hyped-up, right excited. [I] couldn’t wait to go.

For the early embracers, the liberation procedure seemed like a promising opportunity to get better. In the eyes of these participants, Zamboni’s procedure was a credible treatment for MS that made intuitive sense to them. For instance, one participant thought that the Zamboni procedure made sense to her because it was explained in the W5 episode (Favaro & St. Philip, 2009) as being akin to a clogged sink that needed to be drained. She voiced the following:

The way they were explaining it . . . like a clogged sink and the blood was clogged to your brain and your blood was probably doing the damage. It made sense what they were talking about.

For the other early embracers, the liberation procedure seemed logical according to their knowledge about coronary angioplasty. As expressed in the following quotation, they did not see the difference between coronary angioplasty and angioplasty of the jugular and/or azygos veins:

That’s like if people got a blockage in your heart . . . that’s done. What’s the difference with a blockage in my neck [that] they can’t touch my neck?

The fact that the liberation procedure was the topic of scrutiny for the reputable W5 investigative journalists lent legitimacy to the procedure. Indeed, several of the early embracers interpreted the information provided in the W5 program (Favaro & St. Philip, 2009) as “there’s enough science behind this.” One participant wondered the following:

They’re saying there’s not enough study done about it. What [more] do they need? . . . They know that the blockage is there. They know that the MS patients got them.

While contemplating how researchers were stating that the research performed till date on the procedure was inconclusive, a participant commented as follows:

I think they should let it go and pass through regardless because it’s bettering people’s lives.

On further exploration of their understanding of Zamboni’s theory regarding the etiology and treatment of MS, the early embracers offered vague explanations of both the theory and the Zamboni procedure itself. This was the case even though all but two of them had already undergone the procedure. An early embracer narrated the following:

On the way down to the OR, . . . I was getting dizzy, and on the way back up, . . . I wasn’t getting dizzy at all. I guess it was the iron floating around in the brain cavity or whatever. That’s what the fellow [Zamboni] who came up with it said . . . The iron in the brain cavity is a bad thing. So I’m just assuming maybe that’s what happened. They opened up the cavity [and] the pressure came off my head . . . I don’t have like pressure on my head anymore.

All five female early embracers were convinced that the liberation therapy would relieve some of their MS symptoms, but it would work better for those who were newly diagnosed or had mild symptomatology. To them, it was difficult, if not impossible, to heal some of the damage done to the body. As a case in point, one woman deemed the following:

Over the years, there’s been some damage done that probably won’t come back.

Conversely, the male early embracers were under the impression that the liberation procedure would work equally well for everyone, because, in effect, it was a cure. For example, when asked to clarify whether or not he believed that the Zamboni treatment was a cure, one man responded in the following way:

Interviewer (I): Do you think it’s a cure?

Participant (P): Everyone says no, but I think personally myself, I think yes. I really do think yes . . . I think that someday I’m just going to run . . . That’s how much I believe in it.

The liberation procedure was a promising opportunity for the early embracers in another way. The procedure not only appeared to be a logical, legitimate therapy for MS but also seemed to be a simple and safe treatment. The early embracers believed that the procedure was completely risk free, with the exception of two participants, who pointed out that all medical procedures have inherent risks. Nevertheless, they judged any risk of adverse events associated with the liberation procedure to be very small. The other early embracers took it for granted that there was no risk of adverse outcomes. One participant asserted,

It’s not going to harm you . . . It’s got no complications . . . The procedure is a very simple procedure. There was nothing to it.

They thought this was particularly true if a stent was not implanted. This was exemplified in the following quotation:

So I don’t see any risks. Like I’m pretty sure they know what they’re doing . . . Now, if I had to get stents, maybe I’d be a bit nervous, but I don’t see any risks, and if anything is going to come out of this, it’s going to be positive.

#### Desperately trying to ascend to a better quality of life

Together with sharing similar views of, and reactions to, the portrayal of the liberation procedure as a novel treatment for MS, the early embracers all had advanced or unstable MS disease. In the preprocedural period, among the seven early embracers, two had difficulty in walking unassisted, two used a cane, and three used a wheelchair or motorized scooter. Given their relatively advanced stage of disease or unstable situation, the early embracers felt that they were desperate for a cure or at least something to relieve their suffering or to reverse their disease to an earlier state, at which they were more independent and coped better with MS. Participants illustrated these points with comments such as the following:

If I could . . . go back to the way that I was 5 years ago, it would be worth every penny

and

After so many years, you’d almost try anything to give you a bit of a break . . . After a while [of] suffering so much, any kind of hope at all you’re going to try it.

In the midst of a fast downward spiral or feeling at rock bottom, the liberation procedure represented hope or a chance for a better quality of life and, at a deeper level, life itself. The early embracers reckoned they had “nothing to lose” in their attempts. For instance, one person shared the following comment:

I had nothing to lose . . . I am grabbing at straws, but I looked at this as a better quality of life . . . To me, I’m right down here at the bottom, and the only way to go is up!

Another early embracer’s account was as follows:

You got nothing to lose . . . In the last couple of years, my health was just going downhill, downhill, and downhill. Like the first . . . 12 years when I had MS, I never used to have a cane, but [since] the last couple of years, I started dragging my feet . . . and everything started going downhill . . . [I would do] anything for hope and hope was there and I wanted to go. Yeah, I was starting to trip over my feet worse, and . . . I started to hold onto that cane for dear life.

The early embracers thought that nonstent angioplasty of the jugular and/or azygos veins was relatively safe. Nevertheless, when posed with hypothetical questions about the extent of risk they would be willing to take, it was evident that these participants would gamble their lives for an opportunity to get better. An early embracer recounted,

They’ve had a couple of deaths too. I’ve heard that . . . but I’d take a chance . . . Somebody said . . . “Would you take a chance?” I said, “Indeed I would!”

In addition, take, for instance, the following exchange between one early embracer and the interviewer, who tried to determine the extent of mortality risk that the participant would take to undergo the experimental procedure:

I: What about if they said there’s a chance that you’ll die?

P: Oh yeah. No, I don’t think it would deter me.

I: How much of a risk are you willing to take in terms of numbers, fifty–fifty?

P: Oh, yes definitely. You know, I really don’t think I’d hesitate.

On a similar note, another early embracer estimated that the worst case scenario that could possible arise from undergoing the liberation procedure was not death or a stroke, but the continuation of his life in a wheelchair. Echoing these sentiments, another early embracer did not fear the procedure itself; rather, he was more afraid of the consequences of not proceeding with it. All of the early embracers who mentioned whether or not they would have agreed to a stent indicated that they would have given consent for it, despite their knowledge of the risks associated with their use.

All but two of the early embracers quickly set about to undergo the liberation procedure in Poland, Costa Rica, or the United States. In the case of the two exceptions, the participants could not afford the high cost of traveling abroad for the procedure, although if they could, they would “jump at the chance,” as phrased by one of these two early embracers. One participant in this study who underwent the Zamboni treatment paid $6,300 for the procedure alone. Others noted that they incurred between $14,000 and $20,000 in total expenses to undergo the procedure outside of the country.

#### Having no regrets

At the time of the interviews, the early embracers who underwent the liberation procedure (*n* = 5) reported several favorable postoperative outcomes. All of them gave credit to the procedure for improving their overall mental well-being. Furthermore, all of them either mentioned better motor function (*n* = 4) and/or improved sensation in their extremities (*n* = 4). Two of these early embracers maintained that their circulation was enhanced, whereas another two individuals cited more energy and less fatigue. These participants also noted greater bladder control (*n* = 1), less vertigo (*n* = 1), and fewer headaches (*n* = 1). Among the three early embracers who received preoperative disease-modifying therapies, one stopped taking the medications after the liberation procedure.

Two early embracers reported unfavorable outcomes. One developed a thrombosis that was likely induced by the liberation procedure. In the second case, the participant attributed a relapse of symptoms after the first procedure to the fact that he ultimately never received a stent, even though the doctor performing the procedure tried to insert one. The participant subsequently underwent the procedure again, being the only early embracer who underwent the procedure twice.

All of the participants in this study saw the W5 program (Favaro & St. Philip, 2009), either on television or the Internet. The program featured a man who was formerly in a wheelchair but could suddenly walk perfectly after the liberation procedure. Despite their less-than-miraculous results, the early embracers were generally very pleased with the procedure and their outcomes. None of them regretted her or his decision to undergo the liberation procedure, and all said they would highly recommend it to others. Because of the presence of less stiffness in her hands and slightly better balance compared with her condition before the procedure, one early embracer proclaimed the following to other individuals with MS:

Oh my God! I wouldn’t change my decision . . . Yes, go and have it done because I’m one hundred percent [better] to what I was . . . Mine worked perfect.

The early embracers surmised that exercise was necessary to walk again without difficulty or assistance. In fact, the two early embracers who reported the greatest physical improvements began working out actively on their return to Canada. Another early embracer figured she would be able to walk again with physiotherapy after medical treatment for her thrombosis.

#### Late embracers

In the wake of the W5 program (Favaro & St. Philip, 2009), some of the embracers of the procedure did not readily decide to have Zamboni’s treatment. Two participants fell into the category of late embracers because of the later timing of their decision to proceed with Zamboni’s treatment. We identified three themes in the stories of the late embracers: greeting the liberation procedure with skepticism, a last-ditch effort, and risking everything (see Table 1).

#### Greeting the liberation procedure with skepticism

In contrast with the hope and excitement experienced by the early embracers, the two late embracers were skeptical as they watched or heard about the W5 show (Favaro & St. Philip, 2009). Skepticism emerged, for instance, in the following quotation:

They [supposedly] had the big cure . . . I was a big skeptic.

For months, the late embracers were staunchly opposed to the liberation procedure. They were well aware of glorified stories of so-called cures in the past, and, at first, Zamboni’s theory and therapy failed to make any sense to them. A late embracer described her initial reaction to the W5 broadcast (Favaro & St. Philip, 2009) as follows:

I watched the W5 special and . . . I was thinking, “No, there is something not right about this.” I spoke to . . . Dr. [name] about it . . . because it just didn’t make sense to me. . . . It wasn’t logical.

The late embracers encountered red flags warning them against undergoing the procedure, which fueled their skepticism. For example, because of her university background, one late embracer was cognizant of the fact that the theory and accompanying procedure lacked scrutiny and study by the global scientific community and that consequent, rigorous research laid ahead. In the following passage, a late embracer remarked about red flags on which he stumbled:

His wife was . . . after having it done and . . . I was talking to him about it. [He said,] “Wife just came back and she’s going down again now.” Okay, red light there. “Why is she going down again?” “Well, they kind of collapsed in again. She’s going to get them blown out again.” Okay, red light.

#### A last-ditch effort

For the late embracers, the shadow of doubt cast over the liberation procedure did not dissipate for months. When faced with a pivotal situation, they changed their skeptical stance toward the procedure. One late embracer started to hear too many good stories to ignore, and he came to the realization that he was just dying and living a hellish existence. He explained the following:

[I was] just getting sicker and sicker and just watching . . . life go on . . . I just couldn’t take it no more. [It’s] do or die because it’s a hellish existence . . . The drugs [are] . . . doing nothing. You know, it’s not slowing [MS] down . . . I’ve got a bit of hope now. I’m not just dying . . . There’s nowhere to go but up anyway. Instead of waking up worse every day, now I’m getting a bit better.

The other late embracer’s circumstances included a sharp turn for the worse in her health status. As illustrated by the following statements, she found herself in the same desperate, rock-bottom position as the other embracers, where there was nowhere to go but up. The liberation procedure gave them a glimmer of hope to either stave off death or enhance their quality of life. One late embracer provided these insights:

My life had spiraled downward . . . I was in a wheelchair. I had very little quality of life at that point . . . I am still alive, but . . . I was in bed a lot . . . The fatigue was just so debilitating . . . I was just there. So everybody was saying to me, “Give it a try . . . What do you have to lose?” And finally . . . one day I just said, “You know what? I really don’t have a whole lot to lose. So why not?” . . . It comes down to desperation.

On reflection, the late embracers came around to the idea of undergoing the liberation procedure. Neither of them thought it was a cure for MS, but they believed that it could alleviate some of their MS symptoms. Similar to the female early embracers of the procedure, the late embracers surmised that once some of the damage was done, it was permanent. The late embracers chimed in with their early counterparts in calling for publicly funded health care coverage for the procedure to treat CCSVI, regardless of whether or not the individual had MS. One late embracer simply stated the following:

MS related or not, if you have blocked veins, open them.

The late embracers highly recommended the procedure to other individuals with MS. However, they were also convinced that exercise was key to any recovery.

#### Risking everything

In sharp contrast to the belief held by the early embracers that there was little or no risk involved in the liberation procedure, the late embracers perceived that it could be very dangerous and that they were pioneers of the system. Both late embracers knew about the Canadian case of Mahir Mostic, who was in the news because he apparently died from postliberation treatment complications (“Ont. man dies,” 2010). In fact, one late embracer knowingly underwent the procedure in the same facility as Mr. Mostic shortly after his death. She recalled the following:

I went to South America to have that procedure done right at the height of the controversy . . . And it was just touted as being dangerous. I mean [when] I went to Costa Rica . . . after that poor man who died. . . . It was the same facility . . ., the same everything . . . You just get desperate and you take those chances, and you hope to God it . . . [doesn’t] happen to you.

Despite their knowledge of the potential dangers associated with the liberation procedure, including the risk of death, the late embracers divulged that they were willing to accept the risk of undergoing the procedure. In fact, one late embracer ended up undergoing the procedure on two separate occasions and assuming the risk twice because she thought her first procedure was botched. The other late embracer emphatically stated the following:

P: Do or die!

I: So if they told you, “Okay, it’s [a] fifty–fifty [chance] you could die in this procedure or not,” how much would you risk?

P: I’ve been doing nothing but getting worse over 5 years. Yeah, do or die.

On a final note regarding the late embracers, one of them reported an increase in her quality of life after the liberation procedure, an improvement in her gross motor function, and more energy. Together with an improvement in his motor performance, the other late embracer also experienced less paresthesia and “brain fog.”

### Waiters

In this study, six of the participants followed a different decision-making path compared with the early or late embracers of the liberation procedure. On the basis of their experience, we categorized these six participants as “waiters.” The data analysis revealed four themes in the case of the waiters: putting the liberation procedure under the microscope, skeptically refusing to jump or stay on the bandwagon, not wanting to rock the boat, and waiting for credible research results (see Table 1).

#### Putting the liberation procedure under the microscope

The waiters also took notice of the W5 program (Favaro & St. Philip, 2009) because news of the liberation procedure spread rapidly throughout the MS community. The television broadcast (Favaro & St. Philip, 2009) on the procedure piqued their interest, and they were intrigued by the possibility of a major breakthrough in the treatment of MS. The program (Favaro & St. Philip, 2009) was somewhat compelling to them, and they tended to be swayed toward it.

The W5 program (Favaro & St. Philip, 2009) generated a great amount of hope and excitement among several individuals in the MS community. Early embracers of the liberation theory and procedure quickly took steps toward undergoing the procedure, if possible. Meanwhile, the waiters seriously considered the procedure for themselves. As a matter of fact, two of these participants had booked procedures abroad. At this time, equipped with their postsecondary education, the waiters began to put the liberation theory and procedure under the microscope. For instance, one read and critiqued the original research article by Zamboni and his colleagues (Zamboni, Galeotti, Menegatti, Malagoni, Gianesini, et al., 2009). She explained the following:

In that [research] article I read, it was only for 18 months, and then some of their symptoms came back. And half of the people who had the vein opened, it reclosed as soon as they took it out . . . unless they had a stent put in . . . I read that article so I knew the numbers I was working with . . . That’s not very many people that they tested.

#### Skeptically refusing to jump or stay on the bandwagon

For all the waiters, on closer inspection, skepticism began to creep into their thoughts about the theory and procedure. Their discussions with other individuals, such as health care professionals and other MS patients, sowed seeds of doubt about the procedure. One waiter asserted the following:

I’m not going to hop on board just because everyone else is doing it and hope for the best . . . From the information that I was given from Dr. [name], I just didn’t feel that it was enough to go by to jump on the wagon and say, “Let’s go for it.”

At this point in time, the waiters began to change their minds about the procedure, and the two individuals who had made arrangements for the procedure canceled their plans. One of these two individuals related the following:

[It’s like] snake oil . . . They can say anything works . . . I met this lady [with MS] and [we] talked about the liberation treatment and she said, “I had it done as soon as I was diagnosed.” And I said, “How did it help you?” She said, “Well, now I’m using a walker.” . . . [She was] just hoping to halt the progression of the disease and it didn’t . . . After hearing a bit about it and finding out a bit more information, I said, “This is not as good as they say it’s going to be.” So that’s why I changed my mind.

#### Not wanting to rock the boat

Although they wished it was a cure, the waiters ultimately took the stance of health care professionals and researchers, who cautioned MS patients against undergoing the experimental procedure. From the vantage point of the late embracers and waiters, the liberation procedure entailed a high level of risk, including the risk of stroke and death. However, one distinction between the two groups was that the waiters were not prepared to accept the risk associated with the new procedure. Given that the waiters were comparatively well and their conditions were stable, they concluded that it was best to not “rock the boat,” as summed up by one waiter. These participants used other similar expressions, such as “Don’t fix what’s not broken” and “Leave well enough alone.” The following excerpt elucidates this perspective:

Why am I going to try and fix something right now that’s really not broken because after I got . . . stable, I haven’t had an issue since then, not one flick? So I’m not going to rock the boat to try something.

Having said that, the waiters empathized with the embracers and stated that they would act in the same manner as the embracers if their condition deteriorated. In other words, they could relate to the expression “do or die.” One waiter stated the following:

Oh, there’s no possible way I could have lived the first 6 weeks the way I was, absolutely not. If I . . . had some kind of paralysis, major paralysis, it wouldn’t take me long to try anything because I’m such an active person, and for me to come down to that level . . . would be catastrophic to me.

#### Waiting for credible research results

The waiters took stock of their own situation, and the picture that emerged was one of patience. Unlike the embracers of the procedure, who the waiters deemed had more severe symptoms and required a decision more rapidly, the waiters gathered that they had time to wait for the follow-up research results they craved. They believed that the procedure could be a hoax, and, as exemplified in the quotations that follow, they were comfortable with their final decision to not undergo the procedure, thereby letting others be the proverbial “guinea pigs”:

I was quite comfortable with . . . [the] decision I had made. Myself and my husband . . . said we’d wait and see. . . . [We’d] wait for some [study] results to come in

and

I’ll question on where the [local] study is . . . “How far are they? . . . [Is] there any kind of information?” . . . I’m not going to be the first one to jump on it and say, “Here, use me as a guinea pig.”

## Discussion

Health experts such as Pullman et al. (2013) questioned the rationale of the CCSVI/liberation procedure phenomenon. In the present study, in regard to decision making pertaining to the liberation procedure, we identified two main groups of people: (a) embracers of the liberation procedure and (b) waiters. We subdivided the embracers into early and late embracers. A distinguishing characteristic of the late embracers and waiters was skepticism, whereas desperation was a common core or essence that set both subgroups of embracers apart from the waiters. We discuss both of these defining characteristics herein; however, let us first consider the skepticism, or its absence, in the stories of the participants.

As the late embracers and waiters delved into their narratives, it was clear that skepticism came on the heels of hearing about the liberation procedure sooner or later. Participants raised the idea that the liberation procedure could be a hoax or akin to snake oil, which is a “substance with no real medicinal value sold as a remedy for all diseases” (Pearsall, 2013, “Definition” section, para. 1). This is consistent with the views expressed in other publications on the liberation procedure as a treatment for MS (Oger & Alkhajawah, 2010) and on stem cell transplantation (Dedmon, 2009) and CAM (Bausell, 2007) for a variety of diseases, including MS.

With recognition that further research was needed before the procedure could either receive medical sanction or rejection, the waiters looked on as others became human guinea pigs. Other authors and researchers observed that patients who choose to journey into uncharted medical waters view themselves as pioneers (Caulfield & Zarzeczny, 2012; Rachul, 2011). However, the staff at clinics that offer these unproven treatments have not presented their results for peer review (Caulfield & Zarzeczny, 2012), which could be unbeknownst to the general public. The waiters thought that the liberation procedure could potentially play havoc with their bodies. This finding is not surprising, given the literature on the embodiment of MS. For instance, Gardner and Gronfein (2006) exposed in their research that MS patients armor their bodies in public for protection because they perceive their bodies as “fragile and unpredictable” (p. 83).

In contrast, the early embracers jumped at what they saw as a promising opportunity to get better, with no hint of skepticism in their stories. The W5 program (Favaro & St. Philip, 2009), which featured anecdotal evidence of successful liberation procedures, was very appealing to them. Explanations regarding CCSVI and its accompanying procedure made sense to them, and they deemed that the procedure was, for all intents and purposes, harmless. In a similar fashion, a major finding of Pedersen’s (2013) recent phenomenological analysis of interviews with 46 Danish adults who use alternative medicine was the participants’ belief that these treatments can at least do no harm. Moreover, the appealing nature of the anecdotal accounts is in line with two studies suggesting that MS patients are strongly influenced by anecdotal evidence in their CAM-related treatment decisions (Berkman, Pignotti, Cavallo, & Holland, 1999; Olsen, 2009).

Other research demonstrated that individuals with MS would rather receive health information on MS in general from an “expert patient” (Malcomson, Lowe-Strong, & Dunwoody, 2008, p. 671) or from the Internet, including social media websites (Marrie, Salter, Tyry, Fox, & Cutter, 2013), than from a nurse or other health care professional. Indeed, in a study performed by Rachul (2011) that included MS patients who elected to undergo stem cell therapy, although some of the patients tried to obtain peer-reviewed articles in their search for information, most thought that they were well informed on the basis of testimonials of other patients. Furthermore, psychological research has long informed us that narratives are more powerful relayers of a message than any other format, thus leading individuals to place greater weight on narratives than, for instance, on statistics (Shaffer & Zikmund-Fisher, 2013).

Skepticism might have been absent from the descriptions of the experience from the vantage point of the early embracers for another reason. The belief that sufficient research had already been conducted in support of the liberation procedure surfaced in several of their stories. This might have allayed any skepticism they felt. Other researchers reported the misunderstanding of scientific research and skepticism or criticism of evidence-informed health care among patients elsewhere (Carman et al., 2010; Rachul, 2011).

Goldberg (2011) raised the alarm that science is at a crossroads between fact and fiction. Numerous factors never seen before (e.g., outpourings of public support for scientifically uncorroborated medical practices or even scientific fraud) are currently threatening the integrity of research. Other authors discussed an emerging threat of “Internet-based practice” (Reekers, 2011, p. 128) or the possibility of public revolts against evidence-informed practice if it got in the way of treatments (Carman et al., 2010). Although the Internet and social media platforms might play a larger role in health care in the future, as outlined in Hawn’s (2009) social commentary titled “Take Two Aspirin and Tweet Me in the Morning,” scholars noted several dangers regarding the Internet and social media from a health perspective (Lau, Gabarron, Fernandez-Luque, & Armayones, 2012). For example, researchers implicated YouTube videos in the normalization of self-harming behaviors (Lau et al., 2012).

It is particularly alarming that some educated individuals who occupy powerful positions in society have, perhaps unwittingly, turned their backs on science. Pullman et al. (2013) discussed the case of a Canadian Member of Parliament who apparently equated scientific and anecdotal evidence for the liberation procedure. An older example involved a judge in Italy who ordered doctors at a local hospital to prescribe Di Bella’s treatment, which is an unverified miracle cure for various diseases, including cancer, MS, and Alzheimer’s disease (Abbasi, 1998).

For the early and late embracers alike, desperation lurked behind their decision to undergo the liberation procedure. This finding mirrors research in the area of CAM for MS patients (Hussain-Gambles & Tovey, 2004; Rachul, 2011). An examination of qualitative research on the experience of living with MS provides some clues that could help in explaining these findings. In particular, qualitative research brought to the fore the importance of trying to maintain power (Olsson, Lexell, & Söderberg, 2008), being proactive (Malcomson et al., 2008), and using hope in coping with chronic dread and an unpredictable disease (Kirkpatrick Pinson, Ottens, & Fisher, 2009). Also, the desperation that was rampant in the accounts of all of the embracers and the willingness of the late embracers to accept a perceived high degree of risk are in keeping with the terror management theory (TMT). Studies in the area of TMT have demonstrated that individuals, particularly those with an external locus of control, engage in high-risk behaviors when faced with thoughts of their mortality (Greenberg & Arndt, 2012; Miller & Mulligan, 2002).

In closing, we need to consider the findings of this study in light of some limitations. First, the participants in this study consented to interviews that lasted between 60 and 90 min. There might have been individuals who heard about the study and decided not to participate because of time constraints or other reasons. It is possible that nonparticipants might have experiences different from those of the 15 participants in this study. A second limitation was that the study findings were based on one interview with each participant. Further interviews could have shed a different or better light on the experience. Nevertheless, we acknowledge that a good interpretation is never final; rather, it keeps the conversation going (Jardine, 1992). In this spirit, we recommend further qualitative research in this area to continue the dialogue. Together with the findings of this study, this might translate into reflective practice that can better equip nurses and other health care professionals to help MS patients in making informed decisions about the liberation procedure.
